# The many origins of extremophile fishes

**DOI:** 10.1098/rspb.2025.0217

**Published:** 2025-05-14

**Authors:** Chase D. Brownstein, Richard C. Harrington, Olga Radchenko, Thomas J. Near

**Affiliations:** ^1^ Department of Ecology and Evolutionary Biology, Yale University, New Haven, CT, USA; ^2^ South Carolina Department of Natural Resources, Columbia, SC, USA; ^3^ Institute of Biological Problems of the North, Far Eastern Branch, Russian Academy of Sciences, Magadan, Russia; ^4^ Yale Peabody Museum, New Haven, CT 06511, USA

**Keywords:** extremophile, hydrothermal vent, polar, intertidal, fishes, phylogenomics

## Abstract

Extremophiles survive in environments that are considered uninhabitable for most living things. The evolution of extremophiles is of great interest because of how they may have contributed to the assembly of ecosystems, yet the evolutionary dynamics that drive extremophile evolution remain obscure. Here, we investigate the evolution of extremophiles in *Zoarcoidea*, a lineage of over 300 species of fishes that have colonized both poles, the deep sea, and hydrothermal vents. We show that a pulse of habitat invasion occurred across over 20 different zoarcoid lineages within the last 8 million years, far after the origin of their prototypical innovation for surviving in cold water: type III antifreeze protein. Instead, a secondary burst of anatomical, physiological and life history traits and a handful of founder events in extreme ecosystems appear to have propelled zoarcoid diversification. These results decentralize the role of prototypical changes to organismal biology in shaping extremophile radiations and provide a clear example of how a combination of ancient adaptations and recent contingency shapes the origination of lineages in challenging habitats.

## 1. Introduction

Extremophiles are organisms that occupy regions where thermal, chemical, pressure and other conditions are intolerable for most life forms [1–4]. Extremophiles provide insights into the process of biological innovation by revealing how life adapts to seemingly inhospitable environments. Hypothetically, organismal diversity must have first accumulated in the extreme chemical and physical conditions of the early Earth [5]. Yet, for non-microbial life, the evolutionary history of extremophiles is far less studied [2,6–8]. Among vertebrates, fishes comprise a large proportion of potential extremophile taxa, and several lineages have heavily modified their physiology to tolerate freezing cold waters [9–14], hydrogen-sulfide rich springs [15]and the hot, heavy metal-rich waters surrounding hydrothermal vents [16].

Studies of the evolution of extreme habitat occupation in animals tend to focus on a handful of radiations with common ancestors that possessed key innovations for life ‘on the edge.’ The canonical extremophile radiation in vertebrates are the notothenioid fishes, which rapidly diversified in the Southern Ocean following the evolution of antifreeze glycoprotein in their common ancestor [13,17–23]. However, the restricted distribution of the notothenioid radiation, as well as the observation that this clade is only one of several that comprise polar marine vertebrate faunas [24], makes it unclear whether their evolutionary dynamics are reflective of the larger factors shaping the rapid diversification of polar vertebrates [25] or the accumulation of extremophiles generally.

Here, we employ genome-scale data for another lineage of marine perciform fishes, *Zoarcoidea*, to examine the macroevolution of extremophiles. This clade, which includes eelpouts, pricklebacks, gunnels and wolffishes [26–30], is one of the fastest diversifying lineages of marine fishes [25] and has colonized and diversified in numerous extreme habitats, including freezing cold polar and subpolar waters [24], marine ecosystems below 1000 m depth [9,31] and hydrothermal vents [16,32,33]. Along with the taxonomically problematic snailfishes [34] and the notothenioids, zoarcoids are one of the three most species-rich lineages of fishes in the Antarctic [24], Arctic [35], and adjacent oceans. Some intertidal and nearshore zoarcoids, such as the rock gunnels in *Pholis* and monkeyface prickleback *Cebidichthys violaceous*, have also developed the ability to facultatively breathe air [36,37], and species of eelpouts in the genera *Pachycara* and *Thermarces* are key large-bodied predators of hydrothermal vent faunas [33,38]. These factors highlight zoarcoids as a promising model system for understanding the origin of extremophiles.

Our reconstruction of the macroevolution of zoarcoid fishes shows that clades adapted to extreme conditions originated long after the origin of the prototypical zoarcoid key innovation for extreme habitats: type III antifreeze protein (AFP) [9]. The pulse of extreme habitat invasion we observe in zoarcoids from 5 million years ago to the present appears to have been fueled by recent founder-event colonization and associated with anatomical, physiological and life history innovations that originated far after type III AFP appeared. These results challenge the notion that extremophile diversity results from the rapid colonization of extreme habitats following the acquisition of relevant traits and emphasize the role of contingency in shaping macroevolution in hostile environments.

## 2. Methods

### (a) Systematics

In this study, we follow the systematic approach of the *PhyloCode* [39] as recently applied to ray-finned fishes by Near & Thacker [29]. As such, we follow the nomenclature presented by Near & Thacker [29] for all lineages we discuss in this manuscript.

### (b) Ultraconserved element sequencing and dataset assembly

To comprehensively sample living zoarcoid diversity and infer the position of *Zoarcoidea* among eupercarian fishes, we produced a ultraconserved element (UCE) sequence dataset for 164 individual fishes representing 108 species of *Zoarcoidea* and 31 non-zoarcoids. We included representatives of all major eupercarian orders *sensu* Near & Thacker [29] that were resolved in the UCE phylogeny generated by Ghezelayagh *et al*. [30]. In total, we sampled all major zoarcoid clades except for *Eulophiidae*, which we resolved as the sister lineage of a clade containing *Anarhichadidae* and *Zoarcidae* based on maximum likelihood phylogenetic analysis on the IQ-TREE [40] web server [41] of published Sanger-sequenced nuclear gene data (electronic supplementary material, figure S4). We followed previously published methods [30,42,43] for extracting DNA from tissues using the DNEasy Blood and Tissue Kit, quantifying DNA per extraction using a Qubit fluorometer (Life Science Technologies), confirming high-weight DNA isolation using gel electrophoresis, shearing approximately 500 ng DNA per sample using a QSonic Q800R3 sonicator to produce 300−600 bp long fragments, and prepping libraries using Kapa HyperPrep kits (Kapa Biosystems) and Illumina TruSeq iTru5 and iTru7 adapters [44]. We conducted 150 bp paired-end sequencing on Illumina NovaSeq platforms on pooled libraries and used phyluce−1.7.8 to process raw reads, assemble sequences *de novo*, extract UCEs from genomes of zoarcoids publicly available on GenBank (electronic supplementary material, table S1), and align these UCEs with contigs from previous studies [30,43] to form a 75% complete matrix of UCE sequences. Finally, we used CIAlign [45] with default parameters to identify and remove chimaeric UCE sequence alignments. The final sequence dataset comprised 994 UCEs sampled for 164 individuals.

### (c) Maximum likelihood phylogenetic analyses, species tree inference and nodal support

We used IQ-TREE2 [46] to infer maximum likelihood phylogenies for our UCE dataset under three partitioning schemes: concatenated, treated as a single partition; concatenated, with a partitioning scheme based on the results of partition searching implemented using PartitionFinder 2 [47], and individual gene tree estimation. In each case, we used ModelFinder Plus [48] to infer best-fit models of nucleotide evolution for each gene tree or partition and inferred nodal support using 1000 ultrafast bootstraps. We used the individual gene trees to produce a species tree under the multispecies coalescent model implemented in ASTRAL-III v. 5.7.8 [49]. Next, we used IQ-TREE 2 to estimate gene and site concordance factors, which measure the proportions of decisive gene trees and sites that resolve the same given branch in an input reference tree (here, the phylogeny produced using the concatenated alignment). We used custom scripts (http://www.robertlanfear.com/blog/files/concordance_factors.html) to plot concordance factors and examine their relationships to one another, branch lengths and bootstrap support values. We also used custom Python scripts [50,51] to infer the positions of anomaly zones (regions in the species tree where some gene trees resolve alternative relationships and with higher support than the relationships in the species tree) along the phylogeny we produced using the 994 gene trees in ASTRAL-III.

### (d) Time calibration of zoarcoid phylogeny

We used BEAST2 [52,53] and a newly compiled dataset of six non-zoarcoid and five zoarcoid fossil calibrations to conduct a Bayesian node-dating analysis using our UCE dataset. Previous time-calibrated phylogenies of zoarcoids have employed only a handful of fossil calibrations with loose constraints due to the poor placement of described zoarcoid fossils among living species [28]. Recently, however, new fossils of the genus *Zaprora* [54], an indeterminate lumpenid [55] and an indeterminate anarhichadid [56] have been published from the Neogene of Asia, providing essential information on the timescale of zoarcoid evolution. We used the BEAUTi terminal [52,53] to prepare xml files for analysis in BEAST 2.6.6. We constructed three xml files using three sets of 50 randomly sampled UCE sequence alignments. In each case, we used a general time reversible (GTR) model with the+G gamma among-site rate variation distribution parameter, a relaxed log-normal molecular clock, and the BEAST2 implementation of the fossilized birth–death (FBD) model [57]. For the FBD model, we specified the rho parameter, which is the proportion of living species sampled, as 0.25 following the count of living zoarcoids given in Eschmeyer’s Catalogue of Fishes in September of 2024 (https://www.calacademy.org/scientists/projects/eschmeyers-catalog-of-fishes). We set the origin prior to 83.6 Ma, which is the age of †*Gasterorhamphosus zuppichini*, the oldest known definite member of *Syngnathiformes* [58] and thus the minimum age of the most recent common ancestor of *Pelagaria* (*Syngnathiformes*+*Scombriformes*) and *Eupercaria*. The use of this extinct species as a calibration for the MRCA of crown *Eupercaria* is non-ideal, but results from the absence of fossils with clear affinities to clades within *Percomorpha* known from the Mesozoic [30,59,60]. Because of this, we specified wide bounds on the origin prior: 66.02 Ma, the age of the Cretaceous–Palaeogene transition and thus the maximum age of the oldest perciform-bearing horizons [59], was set as the lower bound, and 93.9 Ma, the Cenomanian–Turonian boundary and the minimum age of acanthomorph-rich marine strata that lack percomorph representatives [59,61–63], was set as the upper bound. We set the diversification rate prior to 0.05, the approximate background net diversification rate of acanthomorphs [30], with bounds of 0.00 and 1.00. For each fossil calibration, we used a monophyletic lognormal MRCA prior to that we adjusted so that 97.5% of the distribution fell before the age of the fossil. We fixed the topology to that recovered in our IQ-TREE analyses of the concatenated UCE dataset, as this approach successively replicated relationships among non-percomorph eupercarians found in previous analyses [30,43] but does not change relationships among zoarcoids relative to the ASTRAL-III phylogeny except for the relative positions of a handful of species in *Opisthocentridae, Pholidae* and *Zoarcidae* that have poorly supported relationships in the ASTRAL-III tree. For each UCE set, we ran MCMC chains three times independently over 500 million generations with a 100 million generation pre-burnin. We checked for convergence of the posteriors and effective sample sizes values over 200 using Tracer v. 1.7 [64], combined the top 10% of posterior tree sets in LogCombiner v. 2.6.7 subsampling every 5000 generations, and summarized them in a maximum clade credibility tree with median node heights in TreeAnnotator v. 2.6.6.

### (e) Diversification rates

We estimated diversification rates along the time-calibrate phylogeny using two methods: BAMM 2.5.0 [65,66] and the CoMET model [67] as implemented in the R package TESS [68]. First, we pruned the phylogeny such that only *Zoarcoidea* and its sister clade *Gasterosteidae* were included in order to test whether zoarcoids show a diversification rate shift at the MRCA of the crown clade. BAMM uses reversible-jump Markov chain Monte Carlo to detect shifts in diversification and estimate diversification through time, whereas TESS CoMET uses compound Poisson process models to estimate instantaneous speciation and extinction rate shifts and mass extinction times across the phylogeny [67,68]. For both analyses, we set the sampling fraction to 0.25, the proportion of living zoarcoid and gasterosteid species sampled according to Eschmeyer’s Catalogue of Fishes in September of 2024 (https://www.calacademy.org/scientists/projects/eschmeyers-catalog-of-fishes). For the BAMM analysis, we forced the phylogeny to be ultrametric using the R package ape [69] and generated starting priors using BAMMTools [65]. We ran BAMM for 50 million generations and assessed convergence using BAMMTools in R. For the TESS-CoMET analysis, we inputted 1 for the prior number of mass extinction events to account for the Eocene–Oligocene Mass Extinction. We set prior speciation and extinction rates to 0.1 and 0.02 following acanthomorph-wide estimates [30]. We set global survival probability to 90%, as approximately nine out of 10 marine species survived the Eocene–Oligocene Mass Extinction [70–72]. We ran TESS-CoMET for 10 million generations with a 30 000 generation pre-burnin and confirmed ESS values >200.

### (f) Habitat ancestral state and historical biogeography reconstructions

We conducted ancestral state reconstructions of depth and geographic region using the R packages phytools [73] and BioGeoBears [74]. For the ancestral state reconstruction of habitat, we classified depth preference into three states following one of the classifications presented in Friedman & Muñoz [31]: shallow (<300 m), intermediate (300−1000 m) and deep (>1000 m). Depth ranges per biogeographic area are summarized in electronic supplementary material, figure S5. We collected maximum depth records from the literature and the FishBase data repository and conducted ancestral state reconstruction using the stochastic mapping function in phytools over 1000 simulations. We then plotted these along the phylogeny and recorded the ages of clades with MRCAs inferred to have undergone transitions among depth states. For transitions that occurred at tips, we took the mean of the Recent (0 Ma) and the median MRCA age of the species undergoing the state transition and its living sister to account for transitions occurring anywhere along the branch.

In order to infer the historical biogeography of zoarcoids, we coded species in our dataset for the four latitudinally bounded regions presented in Hotaling *et al*. [28] based on data from FishBase: Arctic, Northern Temperate, Equatorial, Southern Temperate and Southern Oceans. We confirmed that the input phylogeny, which we pruned to only include zoarcoids, was ultrametric and contained only positive branch lengths as required by BioGeoBears, ran ancestral state reconstructions under three models of historical biogeography with and without the +j jump dispersal parameter (Dispersal-Extinction-Cladogenesis (DEC); Dispersal-Vicariance-Like (DIVALIKE); a Bayesian model (BAYAREALIKE)), compared model fit using log-likelihood and AIC scores (electronic supplementary material, table S2), and ran biogeographic stochastic mapping (BSM) on the best-fit model to estimate the type and number of different biogeographic events along the phylogeny via simulation. For this analysis, we selected 100 as the maximum number of maps to attempt, 50 as the goal, and 400 attempts per branch. After BSM analysis was completed, we extracted the number of cladogenetic events and plotted them against the corresponding ages of nodes along the phylogeny to visualize changes in mode of biogeographic change over time.

### (g) Body shape evolution

We used data from the FishShapes v.1 database [75] of eight measurements relevant to body shape and size to infer patterns in the evolution of body shape in zoarcoids. First, we took the natural log of these measurements. Next, we imported the dataset to R for analysis using the packages phytools and geiger [73,76]. We conducted phylogenetic principal components analysis (PCA) on the log-transformed measurements and used plotting tools in phytools, strap [77], and ggphylomorpho [78] to visualize PCA results. We also used phytools to produce traitgrams for standard length and lower jaw length in zoarcoids, as these traits have previously been considered relevant to the evolution of deep-sea fishes [79]. Next, we used geiger to generate a disparity through time plot for the first principal component values from the phylogenetic PCA.

### (h) Evolutionary history of innovations

In order to infer the evolutionary origins of key physiological, behavioural and morphological innovations along the phylogeny of *Zoarcoidea*, we searched the literature for presence/absence data regarding nine discrete traits: antifreeze protein, predatory burrowing, a continuous caudal fin, association of juveniles with scyphozoan jellies, fangs, molariform teeth, sexual dimorphism in heterodonty, ovoviviparity and facultative air-breathing. These traits were selected based on their status as classically important life history and ecological traits (for example, ovoviviparity, air-breathing, antifreeze protein) [9,80–84] or because they have been investigated as characteristic of the ecology or functionally relevant regions of the anatomy of zoarcoid fishes (for example, the presence of different tooth types, the association of *Zaprora silenus* juveniles with jellyfishes) [85–90]. We used the same protocol employed for our ancestral state reconstruction of depth to run stochastic mapping for each of these nine traits in phytools [73]. Next, we used custom scripts to extract the origin times of discrete traits along the phylogeny from the simulated histories we generated using stochastic mapping. We plotted these data using functions from ggplot2 [91].

## 3. Results

**Figure 1.**
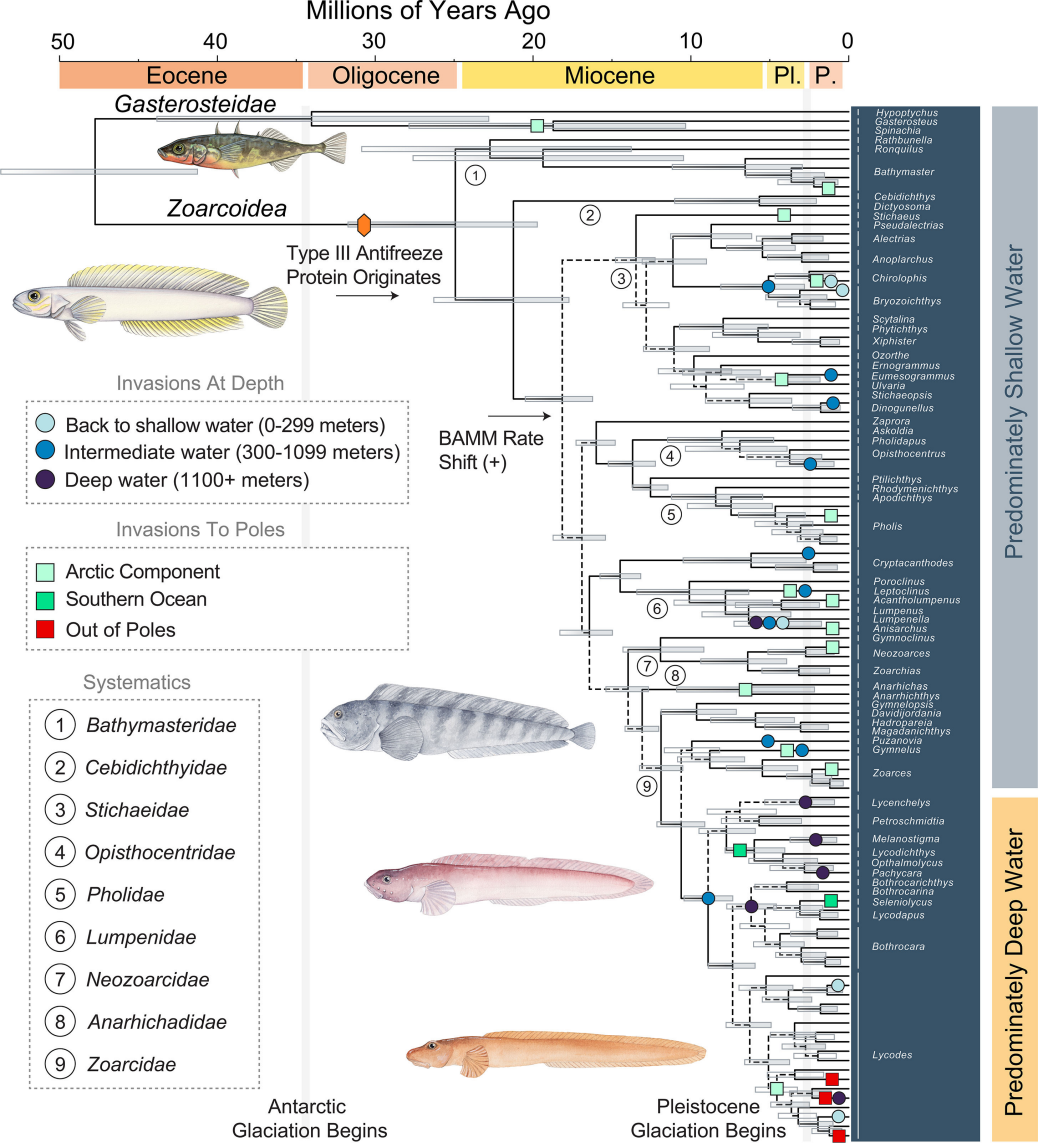
Evolutionary history of extremophile fishes. Time-calibrated phylogeny of *Zoarcoidea* inferred using BEAST 2.6.6 [52,53] on three sets of 50 randomly sampled UCEs and 11 fossil calibrations, with the inferred positions of habitat and depth shifts noted along branches. The time tree, which shows branches with median node age values, was fixed to phylogenetic topology generated from the analyses in IQ-TREE2 where the 994 UCEs were concatenated. Bars at nodes indicate 95% highest posterior density intervals for divergence times, clear bars indicate ultrafast bootstrap values less than 100%, and dotted branches are those with 10 or more anomalous branch pairs. Illustrations are by Julie Johnson (https://www.lifesciencestudios.com)

The phylogeny of zoarcoids has only been investigated a handful of times and has yet to be analysed using a comprehensive genome-scale dataset [9,28,30,92,93]. We recovered a total of 994 ultraconserved elements for 165 individual fishes (figure 1, electronic supplementary material, figures S1–S3) including 154 perciforms and 133 specimens of *Zoarcoidea* representing 108 species (108/429; 25.2% of zoarcoid diversity; [94]) across all zoarcoid families and subfamilies except the species depauperate *Eulophiidae* [28,93]*,* which we find is sister to the clade containing *Anarhichadidae* and *Zoarcidae* using Sanger-sequenced nuclear genes (electronic supplementary material, figure S4) in agreement with previous studies [28,92,93]. Phylogenies generated from our UCE dataset agree with previous analyses [9,28,30,92,93] in several important ways: we infer that the ronquils (*Bathymasteridae*) are the living sister lineage of all other zoarcoids and resolve the traditional *Stichaeidae* as paraphyletic. Lineages classically placed in *Stichaeidae,* including *Cryptacanthodes, Cebidichthyidae*, *Lumpenidae, Neozoarcidae* and *Opisthocentridae*, are resolved as successively nested pectinate lineages in the phylogeny with gunnels (*Pholidae*), prowfish (*Zaprora silenus*), quillfish (*Ptilichthys goodei*) and a clade containing wolffishes (*Anarhichadidae*) and eelpouts (*Zoarcidae*). We also consistently resolve a clade that includes sticklebacks, tubesnouts and sand eels, which form *Gasterosteidae sensu* [29], as the living sister to *Zoarcoidea*. By time-calibrating the zoarcoid evolutionary tree using a set of 11 fossil calibrations (electronic supplementary material), we infer that *Zoarcoidea* originated around the Oligocene–Miocene boundary (median age: 24.92 Ma, 95% highest posterior density interval (HPD): 19.73, 31.72 Ma) and that all major crown clades had diverged by 10 Ma (figure 1).

We infer high levels of discordance, including disagreement among multispecies coalescent and maximum likelihood phylogenies (electronic supplementary material, figures S1–S4) and the presence of several anomaly zones (regions where a handful of gene trees have strongly supported alternative topologies to the species tree; figure 1) [50,51,95,96], within the major zoarcoid clades *Opisthocentridae, Pholidae*, *Stichaeidae* and *Zoarcidae*. Together with the time-calibrated phylogeny, our inference of anomaly zones in these clades is indicative of rapid successive divergences in their evolutionary history. Particularly notable is the anomaly zone at the base of the deep-sea zoarcid radiation that we resolve is the sister lineage of *Lycodes* (figure 1), which originated in the Late Miocene (median age: 6.18 Ma; 95% HPD: 4.59, 7.72Ma). Ancestral biogeographic and habitat reconstructions reveal that this lineage is one of at least 23 clades that invaded extreme environments such as the polar habitats and extreme deep sea environments greater than 1000 m in the last 8 million years. This exceptional number of habitat transitions in zoarcoids supports the recent inference [31] that this clade may possess some of the highest counts of deep-sea invasions among ray-finned fishes and supports the recent age of the Arctic and Antarctic radiations of true eelpouts [24,28,97,98].

**Figure 2.**
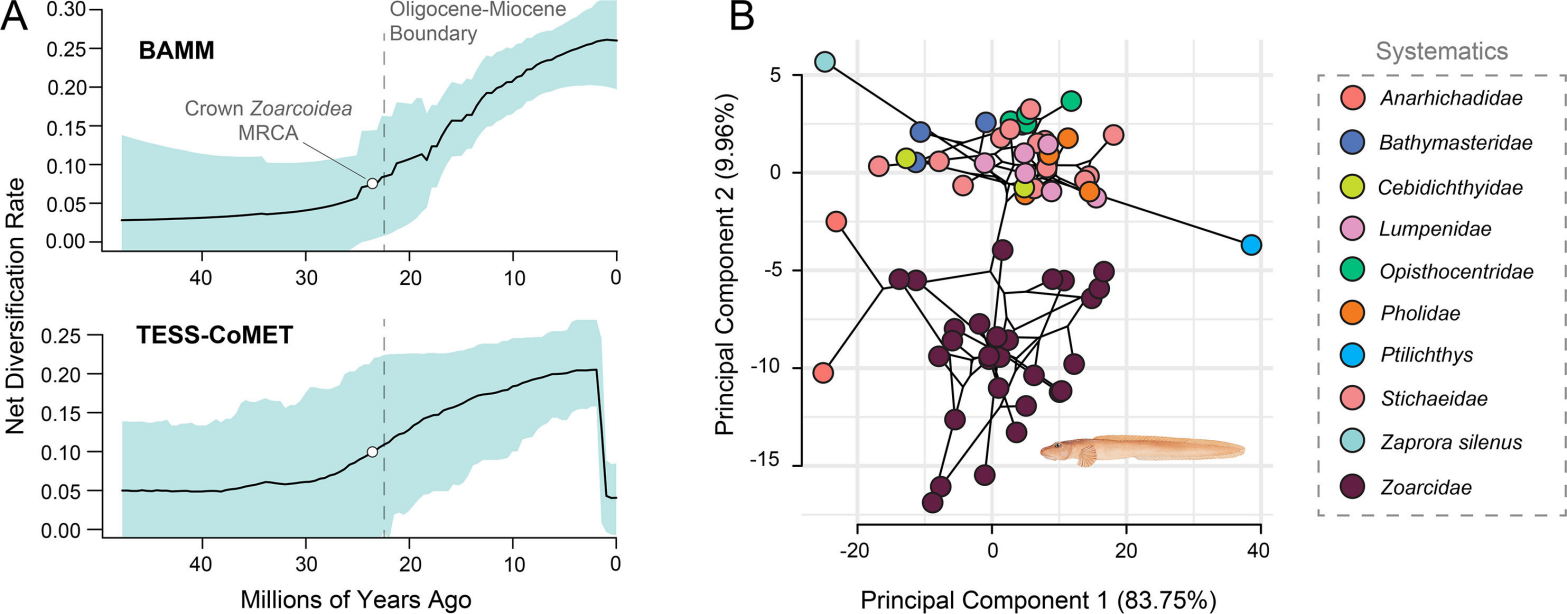
History of diversification and body shape evolution in zoarcoids. (A) Net diversification rate curves inferred using two different methods (BAMM, TESS-CoMET). Black lines indicate mean diversification rate curve, and grey regions indicate 95% confidence intervals. MRCA = most recent common ancestor. (B) Phylomorphospace plot of eight log-transformed continuous body measurements along the time-calibrated phylogeny, with specific clades labelled. Illustration is by Julie Johnson (https://www.lifesciencestudios.com).

The numerous radiations of zoarcoids into cold and freezing waters far postdate the origin of type III antifreeze protein (AFP III) at the origin of *Zoarcoidea* [9,80]*,* which is thought to have provided these fishes with physiological capacity necessary to survive in such extreme climates. These invasions also come halfway through a period of increasing diversification across zoarcoids to the present (figure 2A) and approximately 10 million years after the single positive diversification rate shift most favoured by BAMM at the most recent common ancestor of all zoarcoids besides *Bathymasteridae* and *Cebidichthyidae* (marginal probability = 0.23). Zoarcoids match the classic model of adaptive radiation [99–102] by representing an exceptionally rapid (figure 1; figure 2A) [25] accumulation of ecologically diverse species from a single common ancestor that possessed a canonical key innovation [9], which likely promoted ecological opportunity in novel environments. However, their steadily increasing rate of diversification, along with the non-proximity of the origin of their antifreeze protein to their invasion of deep and polar waters, break from the pattern observed in other classic adaptive radiations [101–103], including notothenioids [17,18], which experience early bursts of species diversification and ecological trait disparity.

To investigate whether patterns of phenotypic evolution in zoarcoids match the expectations of adaptive radiation, we compiled data on body shape and size, as well as potential key physiological, life history, and morphological innovations, across zoarcoid diversity. Our analyses show that whereas zoarcoids do not show early bursts of disparity in body shape or size (67, electronic supplementary material, figure S7), disparity in body shape is concentrated in a handful of lineages (figure 2B; electronic supplementary material, figures S6 and S7). The prowfish *Zaprora silenus* and the quillfish *Ptilichthys goodei* deviate considerably from the conserved body shape of the grade that formerly formed *Stichaeidae*, and the wolffishes and true eelpouts markedly expand the body shape morphospace occupied by zoarcoids as a whole (figure 2B; electronic supplementary material, table S3). Our PCA shows that *Anarhichadidae* and *Zoarcidae*, which include many of the major deep sea and polar invasions that took place within zoarcoids, accessed a novel region of morphospace corresponding to an elongated body shape with a minimally differentiated tail (figure 2B; electronic supplementary material, table S3).

**Figure 3.**
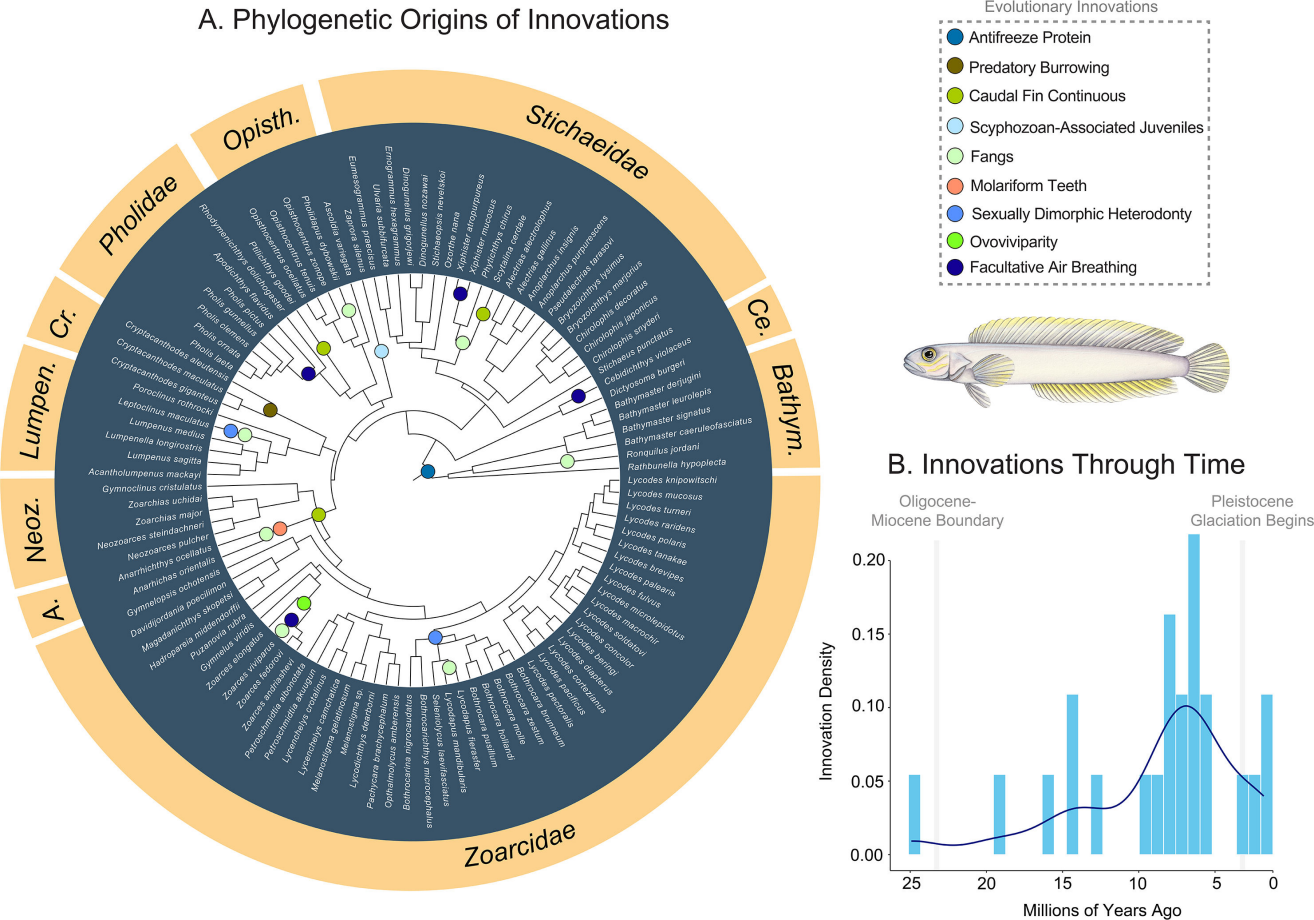
History of key innovations. (A) Time-calibrated phylogeny of *Zoarcoidea* inferred using BEAST 2.6.6 on three sets of 50 randomly sampled UCEs and 11 fossil calibrations, showing origins of key innovations on the phylogeny. (B) Density of key innovation origins through time. Illustration is by Julie Johnson (https://www.lifesciencestudios.com).

**Figure 4.**
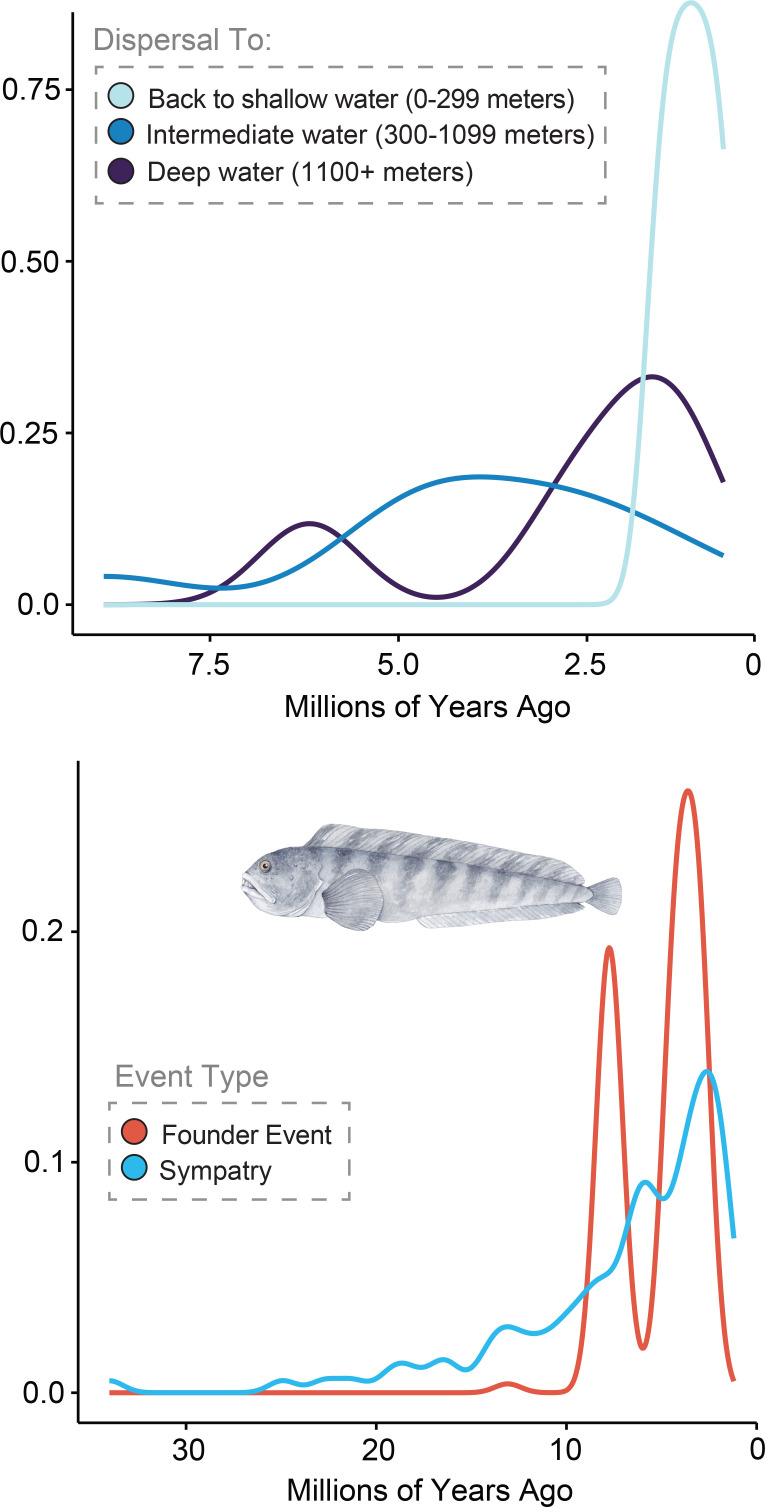
Dispersals at depth through time. (A) Density of dispersals among different depth categories inferred using stochastic mapping in phytools. (B) Density of biogeographic event types through time. Illustrations are by Julie Johnson (https://www.lifesciencestudios.com).

Ancestral state reconstructions of physiological, behavioural, life history and morphological characters considered to be important to the ecology of zoarcoid fishes [37,83–85,89,104,105] similarly show a pulse of innovations occurring between 10 and 5 million years ago. These traits appeared far after the origin of AFP III and between periods of extensive glaciation in the Eocene–Oligocene and Pliocene–Pleistocene [106–111] that shaped the evolutionary history of other cold-adapted [13,18,19] and deep-sea [42,79] fishes, but largely precede the invasion of extreme habitats in many lineages (figures 1, 3 and 4A). Innovations include facultative air-breathing, which we infer convergently evolved in gunnels (*Pholidae*), monkeyface prickleback *Cebidichthys violaceus* [36,37,84], viviparous eelpout *Zoarces viviparus* [81], and twice in *Stichaeidae* [37,112], ovoviviparity, which evolved once in a subclade of eelpouts (*Zoarces*) [82,83,113,114], the extensive association of juveniles with scyphozoans by the prowfish *Zaprora silenus* [85], the multiple origins of fangs across the *Zoarcoidea* (figure 3) and the evolution of molariform dentition in wolffishes (*Anarhichadidae*) [89,90]. These results exemplify the diverse ecologies and life history strategies of zoarcoids and show that, apart from AFP III, many innovations evolved in a pulse (figure 3) prior to a high frequency of invasions into deep sea and polar habitats (figures 1, 3 and 4).

## 4. Discussion

Here, we provide phylogenomic perspective on the evolutionary history of *Zoarcoidea*, which comprises a major component of deep-sea and polar vertebrate faunas and includes numerous lineages that have adapted to extreme environmental conditions. Using ultraconserved elements, we confirm the paraphyly of lineages traditionally classified in *Stichaeidae* [9,28,92,93] and recognize 11 major lineages of zoarcoid fishes (electronic supplementary material, figures S1–S3 and S5) that appeared between 25 and 10 million years ago and subsequently invaded polar and deep water habitats numerous times (figure 1). Several of these family-level clades diverged from each other in rapid succession, a pattern that has left its mark on phylogenies of zoarcoids inferred using genome-scale data in the form of anomaly zones (figure 1).

Our analyses demonstrate that a pulse of invasions to extreme habitats occurred in zoarcoids between 5 and 8 million years ago, including in the most recent common ancestors of least two major radiations, the *Melanostigma–Pachycara* clade in the south and the *Lycodes mucosus-tanakae* clade in the north, which originated within 2 million years of one another and are associated with extensive phylogenetic discordance (figure 1) as often present in rapidly diversifying lineages [30,42,43,96,115–123]. The age of the Southern Ocean zoarcid radiation closely matches with recent estimates of the age of crown *Cryonotothenioidea*, the Antarctic clade of notothenioids, recently obtained from analyses of genomic data [13]. These ages fall just at the end of the Miocene climate transition, which featured global oceanic cooling [124] that even reached the deep sea [125]. However, the ages of these Antarctic fish radiations inferred using genomic data postdate ages inferred using Sanger-sequenced nuclear and mitochondrial genes [17,19,20,25,28] by up to 10 million years. Thus, our time-calibrated phylogeny (figure 1) supports a rapid, recent assembly of the two most species rich lineages of polar fishes [24] over the last 5 million years. Together with preliminary data indicating a Miocene or younger age for snailfishes (*Liparidae*) [30,126], these data show that Arctic and Antarctic marine vertebrate faunas appeared recently in Earth history.

The origin of the canonical key innovation of zoarcoids to cold environments in polar marine habitats, as well as perhaps the deep sea, is far removed from the invasions that produced their current diversity in these extreme habitats (figure 1). Indeed, AFP III was almost certainly present at the MRCA of *Zoarcoidea* [9], far before the first invasions of polar habitats; the oldest invasion into polar oceans by zoarcoids is inferred to occur in *Anarhichadidae*, which enters the Arctic at a minimum of 6.53 Ma (95% HPD: 2.18, 10.9 Ma). In turn, the origination of AFP III, which is analogous to the origin of the antifreeze glycoprotein in notothenioids [13,17], may not fully explain how zoarcoids invaded various extreme environments more than any other clade of ray-finned fishes (figure 1; [31]). Thus, zoarcoids represent a contrasting pattern of diversification in extreme environments to that seen in notothenioids, which rapidly radiated into Antarctic waters following the origination of their antifreeze glycoprotein during the early Miocene, approximately 20 million years ago [13,17,127].

Recent analysis of the evolution of AFP III throughout *Zoarcoidea* based on long-read genome assemblies has shown an expansion of AFP III copies in multiple lineages, including *Anarhichadidae, Pholidae* and *Zoarcidae* [9]. When considered with the evidence for modifications to haemoglobin, including increased dissolved oxygen affinity in the hydrothermal-vent associated zoarcid *Thermarces cerberus* [16] and the presence of multiple distinct hemoglobins in the wolffish *Anarhichas minor* [128], the deep origin of AFP III may represent the start of a cascade of physiological innovations that was associated with, and perhaps facilitated, the evolution of extremophiles in *Zoarcoidea* (figure 3). The recent, phylogenetically restricted origins of some of these innovations (figure 1; figure 3) indicates that extensive sampling of high-quality genomes will be needed to establish the evolutionary history of key proteins involved in physiology and metabolism, such as AFPs and haemoglobin.

The evolutionary history (figures 2B and 3) [9,80] of zoarcoids deviates from expectations of adaptive radiations with early bursts [100,129]. The burst of novelty in this clade that we detect between 10 and 5 million years ago includes numerous physiological, life history and morphological innovations, including the occurrence of ovoviviparity—rare in marine fishes—in species of *Zoarces* [83] and the invasion of new body shape morphospace by true eelpouts and wolffishes (figure 2B). Instead of an early-burst pattern, zoarcoids have experienced increasing rates of diversification to the present (figure 2A) in a matter analogous to other polar-associated deep-water fish clades, such as lanternfishes (*Myctophiformes*) [130]. Indeed, *Zoarcoidea* might best be viewed as an adaptive radiation that was contingent on the presence of both intrinsic adaptations that were secondarily employed for survival in extreme habitats and extrinsic global change like polar oceanic cooling and glaciation [18]. This mélange of intrinsic modifications to key biological systems and the climate change that impacted Earth’s ecosystems over the last 5 million years may account for the sudden, synchronous appearances of extremophile fishes across the globe.

## Data Availability

All data reported in this manuscript is available in the main text, electronic supplementary material available in the FigShare repository associated with manuscript, in the Dryad Digital Repository associated with this manuscript [131], and in the GenBank BioProject associated with this manuscript (PRJNA1236368). Supplementary material is available online [132].
